# Exploring Nomophobia: A Cross-Sectional Study on the Prevalence Among College Students in the Chengalpattu District, India

**DOI:** 10.7759/cureus.71811

**Published:** 2024-10-18

**Authors:** Rajan Edward Daniel Thomas, Harishma Ramesh, Arun Kumar R, Vinoth Gnana Chellaiyan, Sanjutha A

**Affiliations:** 1 Community Medicine, Chettinad Hospital and Research Institute, Chettinad Academy of Research and Education, Chennai, IND

**Keywords:** addiction, mobile phone, nomophobia, students, technology

## Abstract

Introduction

Nomophobia, also known as “NO MObile PHone Phobia,” is a psychological ailment in which people are afraid of losing access to their mobile phones. This study aims to fill this gap by conducting a cross-sectional investigation into the prevalence of nomophobia among college students in the Chengalpattu district.

Methodology

A community-based cross-sectional study was carried out in the selected colleges located in the Chengalpattu district, Tamil Nadu. Undergraduate college students of both genders aged between 18 and 26 years were included in the study. Out of eight administrative blocks in the district, one block was selected by simple random sampling, and out of the eight arts and sciences colleges in the selected block, through the simple random sampling method, three colleges were selected. The line list of eligible students was prepared based on the inclusion and exclusion criteria. A systematic random sampling method was used to recruit the study participants from the line list. A total of 207 students were approached and interviewed using the nomophobia (NMP-Q) questionnaire. Fisher’s exact test was used to find the association between nomophobia and its determining factors. P < 0.05 was considered statistically significant.

Results

Out of 207 participants, seven (3.4%) didn’t have nomophobia, 57 (27.5%) participants had mild nomophobia, 123 (59.4%) participants had moderate nomophobia, whereas only 20 (9.7%) had severe nomophobia. By gender, female students were found to have increased nomophobic characteristics when compared to male students (p<0.041). Also, students with both parents (p<0.003) and those who have reported daytime sleepiness were found to have an association with nomophobia (p<0.018).

Conclusion

Nomophobia is also a growing hazard to students' physical, emotional, and social well-being. As a result, it's critical to optimize students’ utilization of smartphones only for necessities. Concerns have been made that students' lifestyles and quality of life will be seriously damaged if nomophobia is left neglected by physicians and psychologists.

## Introduction

The largest non-drug addiction of the twenty-first century may be mobile phones [1]. Nomophobia, also known as “NO MObile PHone Phobia,” is a psychological ailment in which people are afraid of losing access to their mobile phones [2]. The United Kingdom, or UK, Post Office first used the term "nomophobia" in 2008 when they commissioned YouGov, a UK-based research company, to conduct a study [3]. Based on the Diagnostic and Statistical Manual of Mental Disorders, 4th edition (DSM-IV) standards, nomophobia is defined as a "phobia for a particular/specific thing" [4]. With the release of the very first cell phone in 1983, these devices became indispensable in society. Technology is now ingrained in many demographics, regardless of age, gender, or location, because of developments in cell phones, which vary from basic mobile devices to advanced cell phones and smartphones.

India is a rapidly expanding digital consumer market, with around 560 million internet consumers (almost 41% of the population) in the year 2018 [5]. Compared to the social media consumers in China and the US, Indians spend an average of 17 hours a week on social media sites [6]. The primary advantage lies in the fact it is real-time and portable, offering access to the Internet at any time and from any location. These characteristics have led to increased acceptance and usage of smartphones among adults as well as among kids and teenagers. The majority of entry-level smartphone users in India are students, with half of them being between the ages of 15 and 24 [7]. College students are reported often using their phones for more than nine hours a day, which can become addictive. An illustration of "a paradox of technology" that may both free and enslave people is this. Breaking free from the actual world and becoming enslaved to the digital realm [8].

Nomophobia is characterized by certain symptoms that are present in all cases of access failure (phone loss or misplacing, low battery life, or being in an area without network service). A nomophobe is someone who never turns off their phone, is constantly checking their phone for missed calls and texts, always has their phone with them, uses their phone inappropriately, and misses out on opportunities for in-person interactions in favor of phone contact [9]. In extreme situations, when a phone dies or becomes unusable, users may also experience bodily side effects like panic attacks, dyspnea, sweating, elevated heart rate, discomfort, and so on [10]. Undoubtedly, millions of individuals worldwide experience nomophobia. Given the market's explosive rise for smartphones, it is unsettling to think about how dependent students may become on their mobile devices. As a result, the increasing pattern of excessive use of smartphones jeopardizes public health. Hence, as an initial step to monitor their dependent behavior, this study is directed to measure the prevalence of nomophobia among college students of the Chengalpattu district, Tamil Nadu.

## Materials and methods

A community-based cross-sectional study was carried out in the year 2024 among the colleges located in Chengalpattu district, Tamil Nadu. The study conducted at Chettinad Hospital and Research Institute, Chettinad Academy of Research and Education, Chennai, India, included undergraduate students of both genders from arts and sciences colleges, aged between 18 and 26 years. Students diagnosed with neuropsychiatric illness and under treatment or those having a history of recent episodes of grief were excluded. The study proposal has been approved by the Institutional Human Ethics Committee (IHEC-I/2424/24).

The sample size was calculated based on the study by Mengi A. et al. [11], considering a 40.1% prevalence of nomophobia. With a 95% confidence interval and 7% allowable error, the sample size was calculated to be 188. To account for a non-response rate of 10%, a total of 207 college students were approached and interviewed. Out of eight administrative blocks in the Chengalpattu district, one block was selected by a simple random sampling technique. Out of the eight arts and sciences colleges in the selected block, through a simple random sampling method, three colleges were selected, and the line list of eligible students was prepared. To recruit the study participants from the line list, systematic random sampling was done. The first sample was selected randomly by the lottery method; subsequently, the remaining samples were selected for every 10th sample until the sample size was reached. Out of the 207 students approached, all 207 students responded positively, with no non-respondents.

A pre-tested, semi-structured questionnaire comprising two sections was used to collect the data. The first section consisted of socio-demographic details (Appendix 1) along with a questionnaire on sleep patterns and daytime sleepiness (Appendix 2), and the second section had a nomophobia (NMP-Q) questionnaire to assess the nomophobia level among the study population (Appendix 3). The NMP-Q questionnaire is a 20-item, seven-point Likert scale used to assess the severity of nomophobia. Each question has a score from 1 (strongly disagree) to 7 (strongly agree). Summing up all the responses gives the final score [12]. The final score is used to classify nomophobia into four categories: a score of 20 represents no or absent nomophobia; a score between 21 to < 60 denotes mild nomophobia; a score between 60 to < 100 represents moderate nomophobia; a score of 100 to 140 denotes severe nomophobia.

Data was collected after obtaining informed consent from the participants. Data entry and analysis were done using a Microsoft Excel (Microsoft Corporation, Redmond, Washington, United States) spreadsheet and IBM SPSS Statistics for Windows, Version 21 (Released 2012; IBM Corp., Armonk, New York, United States). Quantitative variables were described in mean and standard deviation, while qualitative variables were described in frequency and percentage. Fisher’s exact test was used to find the association between nomophobia and its determining factors. P < 0.05 was considered significant.

## Results

The mean age of the participants was found to be 19 ± 1.86 years. The majority of the participants were female (65.7%). Only 5% of the population was found married, and 7% were found working part-time after college hours (Table 1).

**Table 1:** Socio-demographic characteristics of the study participants (n=207) *Modified Kuppusamy scale (2023)

Characteristics	Number	Percentage
Age		
18 – 20	176	85.0
21 – 23	19	9.2
24 - 26	12	5.8
Gender		
Male	71	34.3
Female	136	65.7
Marital status		
Married	11	5.3
Unmarried	196	94.7
Part-time employment		
Yes	16	7.7
No	191	92.3
Marital status - Parents		
Married	185	89.4
Divorced	8	3.9
Widow	14	6.7
Socio-economic classification*		
Class I	53	25.6
Class II	55	26.6
Class III	44	21.3
Class IV	55	26.6
Class V	0	0

The mean duration of sleep per day was 6.3 ± 0.4 among the students. On average, 72% of students had about four to eight hours of sleep per day, and 37% reported having daytime sleepiness (Table 2).

**Table 2:** Sleep pattern of the study participants (n=207)

Characteristics	Number	Percentage
Average sleeping hours		
Less than 4 hours	24	11.6
4 to 8 hours	151	72.9
More than 8 hours	32	15.5
Daytime sleepiness		
Yes	78	37.7
Occasionally	73	35.3
No	56	27.1

Out of 207 participants, seven didn’t have nomophobia, 57 participants had mild nomophobia, 123 participants had moderate nomophobia, whereas only 20 had severe nomophobia (Table 3).

**Table 3:** Prevalence of nomophobia (n=207)

Characteristics	Number	Percentage
Absent	7	3.4
Mild	57	27.5
Moderate	123	59.4
Severe	20	9.7

It was found that age, marital status, employment pattern, socio-economic status, and average sleep hours were not associated with nomophobia among students. However, by gender, female students were found to have increased nomophobic characteristics when compared to male students. Also, those students who have both parents and those who have reported daytime sleepiness were found to be nomophobes (Table 4).

**Table 4:** Socio-demographic factors associated with nomophobia (n=207) # Fisher’s exact test; * p < 0.05 – statistically significant

Characteristics	Nomophobia N (%)				p-value
	Absent	Mild	Moderate	Severe	
Age					
18 – 20	7 (100%)	49 (86%)	106 (86.2%)	14 (70%)	0.379
21 – 23	0	4 (7%)	12 (9.8%)	3 (15%)	
24 - 26	0	4 (7%)	5 (4.1%)	3 (15%)	
Gender					
Male	5 (71.4%)	20 (35.1%)	36 (29.3%)	10 (50%)	0.041*
Female	2 (28.6%)	37 (64.9%)	87 (70.7%)	10 (50%)	
Marital status					
Married	0	2 (3.5%)	6 (4.9%)	3 (15%)	0.238
Unmarried	7 (100%)	55 (96.5%)	117 (95.1%)	17 (85%)	
Occupation					
Part-time employment	0	3 (5.3%)	9 (7.3%)	4 (20%)	0.212
Unemployed	7 (100%)	54 (94.7%)	114 (92.7%)	16 (80%)	
Marital status - Parents					
Married	7 (100%)	55 (96.5%)	111 (90.2%)	12 (60%)	0.003*
Divorced	0	0	4 (3.3%)	4 (20%)	
Widow	0	2 (3.5%)	8 (6.5%)	4 (20%)	
Socio-economic classification					
Class I	1 (14.3%)	13 (22.8%)	35 (28.5%)	4 (20%)	0.384
Class II	2 (28.6%)	16 (28.1%)	33 (26.8%)	4 (20%)	
Class III	2 (28.6%)	18 (31.6%)	20 (16.3%)	4 (20%)	
Class IV	2 (28.6%)	10 (17.5%)	35 (28.5%)	8 (40%)	
Average sleeping hours					
Less than 4 hours	0	6 (10.5%)	17 (13.8%)	1 (5%)	0.368
4 to 8 hours	4 (57.1%)	40 (70.2%)	90 (73.2%)	17 (85%)	
More than 8 hours	3 (42.9%)	11 (19.3%)	16 (13%)	2 (10%)	
Daytime sleepiness					
Yes	0	15 (26.3%)	57 (46.3%)	6 (30%)	0.018*
Occasionally	3 (42.9%)	21 (36.8%)	40 (32.5%)	9 (45%)	
No	4 (57.1%)	21 (36.8%)	26 (21.1%)	5 (25%)	

## Discussion

The prevalence of 96.6% nomophobia in the present study coincides with studies by Shaheen et al. in Egypt [13], Setia et al. in India [14], Ayar et al. in Turkey [15], Cain et al. in America [16], and Ma et al. in China [17], which reported prevalences of 100%, 99.8%, 97%, 99.5%, and 83%, respectively. However, few Indian studies had controversial prevalences of nomophobia among their participants, namely 41.8% and 22.3% by Prasad et al. [18] and Ramudu et al. [19]. This difference in nomophobia may be due to the usage of different study tools, different study groups enrolled, and their geographic cultural variations. A gradual decrease in the prevalence of nomophobia has been noted as the age increases. In other words, nomophobia is found to be higher in younger ages (18-20 years). This finding also coincides with an Indian study by Setia et al. [14] and a Turkish study by Yildirim et al. [20].

Students who have both parents living together had a higher association with nomophobia, and those who reported having daytime sleepiness also had nomophobia. Based on the data collected, 89.4% of the study participants have married parents, compared with fewer students with divorced (3.9%) or widowed (6.7%) parents. The extensive use of smartphones and nomophobia may correspond to this variation in parental marital status. The domestic environment of adolescents or young adults with married parents may be more stable, which could either increase or decrease smartphone dependency based on how the family connects and interacts. On the contrary, participants whose parents are divorced or passed away could use cell phones as a way of coping to keep in touch with others or as a way to distract from painful emotional circumstances. The smaller number of participants who had divorced or widowed parents might suggest that those people may use smartphones unconventionally as an outcome of an altered family situation, which could increase their propensity to nomophobia if smartphones become the primary way of gaining social support. In a study by Buctot et al. [21], adolescents experiencing family dysfunction may exhibit higher levels of smartphone addiction and nomophobia. This indicates that the stability and quality of the family environment can impact how young individuals use and depend on their mobile devices.

The reported higher prevalence of nomophobia among students may be due to the following aspects of smartphones: ease of communication, accessibility to information, holding an online identity, and entertainment. Female students were found to have more nomophobia when compared to male students in this study, which again coincides with studies by Shaheen et al. [13], Setia et al. [14], Yildirim et al. [20], Gezgin et al. [22], and Cain et al. [16]. The current study's finding, in accordance with previous studies, illustrated that women had higher levels of nomophobia than men [20,23]. This variation may be credited to the differing methods that each gender uses to communicate. Since they consider smartphones as a tool for emotional support and social interaction, women may develop a stronger emotional attachment to devices. Women are significantly more likely than men to spend more time on social media, which could increase the anxiety associated with being cut off. 

Daytime sleepiness was reported among those having nomophobia in this study, which is in line with the results from Peszka et al. [24] and Erten et al. [25], where daytime sleepiness was reported among the studied student population. There have been reports of behavioral and academic problems in teenagers with nomophobia (such as violence, poor grades, and absenteeism) when they encounter persistent sleep disturbance. There has also been a prediction of an increased chance of accidents among these students.

As the prevalence of nomophobia and its determining factors have been studied among the student population, the scope for further research lies in the impact of nomophobia on academic performance, psychological well-being, and depression.

Limitations

The study has been conducted among college-going young adults. This may not represent the burden among young adults in total, who are more prone to nomophobia. The study has not assessed the academic performances of the study population, which may have an impact on nomophobia and vice versa.

## Conclusions

A significant proportion of Indians who use mobile phones suffer from nomophobia, a disorder that is increasingly affecting students' emotional, social, and mental well-being. Therefore, in order to reduce these negative impacts, it is important to optimize smartphone usage among students and educate them regarding the harmful effects of excessive usage of smartphones. Implementing awareness programs, promoting healthier habits, and encouraging balanced screen time can help foster more mindful and responsible smartphone use. Concerns have been made that students' lifestyles and quality of life will be seriously damaged if nomophobia is left neglected by physicians and psychologists. If there is not enough awareness or intentional tracking of reliant behavior today, then when things go wrong, it may be long past time to recognize that it was the people who were the slaves of technology rather than the other way around.

## Appendices

Appendix 1

**Table 5:** Socio-demographic characteristics of the study participants

S. No	Characteristics
1	UID
2	Age
3	Gender
4	Religion
5	Marital status
6	Name of the college
7	Education
8	Occupation
9	Parents marital status
10	Father’s education
11	Mother’s education
12	Father’s occupation
13	Mother’s occupation
14	Total family members
15	Monthly family income

Appendix 2

**Table 6:** Sleep pattern

S. No	Characteristics	Responses		
1	On average, how many hours of sleep do you get every night?	a) Less than 4 hours	b) 4 to 8 hours	c) More than 8 hours
2	Do you feel sleepy or tired during the daytime?	a) Yes	b) No	c) Occasionally

Appendix 3

**Table 7:** Nomophobia questionnaire (NMP-Q) Responses: 1: strongly disagree; 2: disagree; 3: somewhat disagree; 4: neither agree nor disagree; 5: somewhat agree; 6: agree; 7: strongly agree

S. No	Question	Response
1	I would feel uncomfortable without constant access to information through my smartphone.	
2	I would be annoyed if I could not look information up on my smartphone when I wanted to do so.	
3	Being unable to get the news (e.g., happenings, weather, etc.) on my smartphone would make me nervous.	
4	I would be annoyed if I could not use my smartphone and/or its capabilities when I wanted to do so.	
5	Running out of battery in my smartphone would scare me.	
6	If I were to run out of credits or hit my monthly data limit, I would panic.	
7	If I did not have a data signal or could not connect to Wi-Fi, then I would constantly check to see if I had a signal or could find a Wi-Fi network.	
8	If I could not use my smartphone, I would be afraid of getting stranded somewhere.	
9	If I could not check my smartphone for a while, I would feel a desire to check it.	
10	If I did not have my smartphone with me, I would feel anxious because I could not instantly communicate with my family and/or friends.	
11	If I did not have my smartphone with me, I would be worried because my family and/or friends could not reach me.	
12	If I did not have my smartphone with me, I would feel nervous because I would not be able to receive text messages and calls.	
13	If I did not have my smartphone with me, I would be anxious because I could not keep in touch with my family and/or friends.	
14	If I did not have my smartphone with me, I would be nervous because I could not know if someone had tried to get a hold of me.	
15	If I did not have my smartphone with me, I would feel anxious because my constant connection to my family and friends would be broken.	
16	If I did not have my smartphone with me, I would be nervous because I would be disconnected from my online identity.	
17	If I did not have my smartphone with me, I would be uncomfortable because I could not stay up-to-date with social media and online networks.	
18	If I did not have my smartphone with me, I would feel awkward because I could not check my notifications for updates from my connections and online networks.	
19	If I did not have my smartphone with me, I would feel anxious because I could not check my email messages.	
20	If I did not have my smartphone with me, I would feel weird because I would not know what to do.	
